# Surface Charge Mapping on Virions and Virus-Like Particles of Helical Plant Viruses

**DOI:** 10.32607/20758251-2019-11-4-73-78

**Published:** 2019

**Authors:** M. V. Arkhipenko, N. A. Nikitin, O. A. Baranov, E. A. Evtushenko, J. G. Atabekov, O. V. Karpova

**Affiliations:** Department of Virology, Lomonosov Moscow State University, Moscow, 119234 Russia

**Keywords:** plant viruses, magnetic nanoparticles, surface charge mapping

## Abstract

Currently, the assembly of helical plant viruses is poorly understood. The viral assembly and infection may be affected by the charge distribution on the virion surface. However, only the total virion charge (isoelectric point) has been determined for most plant viruses. Here, we report on the first application of positively charged magnetic nanoparticles for mapping the surface charge distribution of helical plant viruses. The charge was demonstrated to be unevenly distributed on the surface of viruses belonging to different taxonomic groups, with the negative charge being predominantly located at one end of the virions. This charge distribution is mainly controlled by viral RNA.

## INTRODUCTION

Investigation of the physico-chemical characteristics of plant virus virions, including the charge distribution on their surface, may lead to a better understanding of the molecular mechanisms of infection development in the primary infected cell or during the movement of a transport form of RNA viruses (virions/vRNPs) to neighboring uninfected cells. To date, studies exist that describe the features of surface charge formation in icosahedral viruses [1-3]; isoelectric points of virions with various symmetry types have been determined [4, 5]. According to published data, the isoelectric points of most plant viruses fall within the range of 3.6 to 6.3. At neutral pH values, these viruses have total negative surface charges [4, 5]. However, the surface charge distribution of viral particles with a helical capsid remains largely unexplored. According to our preliminary data, the surface of some virions may be charged unevenly. The infection-induced translational activation of the RNA-containing plant virus genomic nucleic acid may also be associated with the surface charge distribution on the viral particle.

In this work, we propose a method for surface charge mapping in helical virions using fluidMAG-DEAE magnetic nanoparticles (MNPs). We studied the surface charge distribution in helical plant viruses from different taxonomic groups (tobacco mosaic virus (TMV) of the *Tobamovirus *genus and potato virus X (PVX) and alternanthera mosaic virus (AltMV) of the *Potexvirus *genus), as well as in virus-like particles (VLPs) and viral ribonucleoproteins (vRNPs) derived from virion components.

A suggestion of a relationship between either the surface charge distribution in plant viruses or the surface charge heterogeneity throughout the entire virion and the accessibility of encapsidated RNA for interaction with ribosomes and RNA packaging in the coat protein (CP) has not yet been discussed.

## EXPERIMENTAL

**Isolation of viruses, viral RNA, and coat proteins**

PVX and TMV samples were isolated according to [6] and [7], respectively; AltMV was isolated according to [8]. RNA was isolated by a modified phenolic method [9]. PVX and AltMV coat proteins were obtained by salt deproteinization [10]. The TMV coat protein was obtained by the acetate method [11].

**Preparation of TMV and AltMV CP (VLP) repolymers and PVX vRNPs**

TMV and AltMV CP repolymers were prepared using the techniques described in [11, 12], respectively; PVX vRNPs were obtained according to [13].

**Treatment of virions and virus-like particles with RNase A and micrococcal nuclease**

Virions and vRNPs with a final concentration of 0.05 mg/mL were treated with RNase A at the ratio 1 μg of enzyme per 4 μg of the virus. Incubation was carried out for 30 min; the reaction was stopped by placing the samples in ice. Micrococcal nuclease (MN) (50 units of active enzyme per 1 μg of RNA) was added to the samples pre-treated with 100 mM CaCl_2_. The reaction was stopped by adding 250 mM EGTA. tRNA was used as a co-precipitator during RNA isolation from nuclease-treated virions.

**Ultrasonic treatment of TMV virions**

TMV particles were sonicated using an Ultrasonic Processor homogenizer. The treatment was performed at a TMV concentration of 0.05 mg/mL, in ice for 60 s.

**Preparation of virion/virus-like particle (VLP)–magnetic nanoparticle complexes**

Virions/VLPs were incubated with fluidMAG-DEAE magnetic particles (Chemicell, Germany) in an aqueous solution at a final virion/VLP concentration of 0.05 mg/mL for 20 min.

**Transmission electron microscopy and nanoparticle tracking analysis**

Samples were adsorbed on copper grids and contrasted according to the procedure described in [14]. The analysis was performed using the JEOL JEM-1011 and JEOL JEM-1400 electron microscopes (JEOL, Japan) at 80 kV.

Samples in liquid were studied by nanoparticle tracking analysis using a NanoSight NS500 instrument and NanoSight NTA 2.3 software (NanoSight, UK). The particles’ Brownian motion was recorded and processed using the following settings: 10 video recordings 60 seconds long each at a camera level of 14 and a detection threshold of 5. The mean hydrodynamic diameter and particle concentration are presented as a 95% confidence interval.

## RESULTS AND DISCUSSION

Virion surface charges were mapped using fluidMAG-DEAE magnetic nanoparticles (MNPs) (Chemicell, Germany) with a specified hydrodynamic diameter of 50 nm. These MNPs consist of an iron oxide magnetic core and a shell composed of starch functionalized with diethylaminoethyl groups. Due to the positive charge of these groups, the MNPs can be used to map a negative charge on the surface of biological structures. The magnetite core enables detection of the MNP position in a complex with viral particles by transmission electron microscopy (TEM).

The mean MNP hydrodynamic diameter measured by nanoparticle tracking analysis was 72 ± 3 nm. According to the TEM data, individual MNPs were assembled into aggregates of 2 to 20 nanoparticles.

The assembly of TMV virion–MNP complexes was performed at a total negative charge of viral particles. The interaction between MNPs and TMV in liquid was studied by nanoparticle tracking analysis. The mean equivalent hydrodynamic diameter of TMV was 115 ± 3 nm. Addition of MNPs to the virus led to an increase of the mean diameter to 134 ± 8 nm at a constant particle concentration of (1.6 ± 0.1) × 10^14^ and (1.6 ± 0.2) × 10^14^ particles/ml, respectively, which indicated complex formation.

**Fig. 1:**
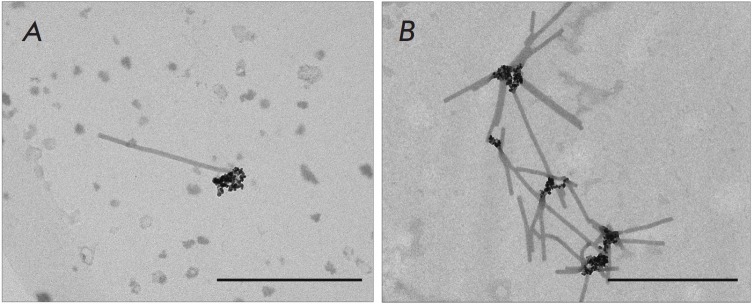
MNPs bind to one end of the native TMV virion. Scale bars are 500 nm

Analysis of TMV–MNP complexes by TEM showed that the magnetic particles effectively formed complexes with TMV bounding to only one end of the virion *(Fig. 1A)*. The so-called "spider" complexes were also detected, which are a group of virions simultaneously interacting by one end with magnetic nanoparticles *(Fig. 1B)*. No complexes of MNPs interacting simultaneously with two opposite TMV ends were detected.

This fact (MNPs interacting with only one end of TMV particles) attracted our attention. In further studies, we used a TMV preparation of 300 nm native particles "broken" by ultrasound (TMV^US^). The mean length of these particles was 149 ± 83 nm.

**Fig. 2:**
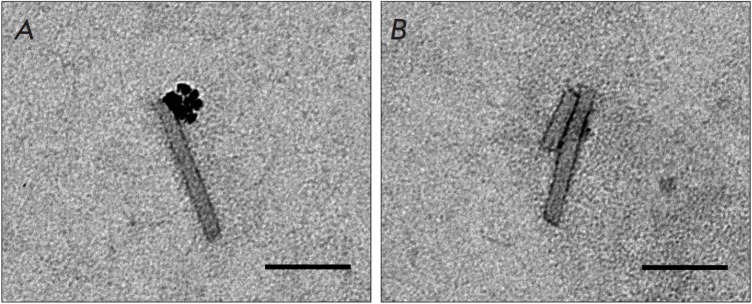
MNPs form complexes with one end of ultrasound-treated TMV (TMV^US^). Scale bars are 100 nm

TEM revealed that MNPs were bound strictly to one end of TMVUS fragments *(Fig. 2A)*, like in the case of native virions *(Fig. 1A)*. However, a certain amount of TMVUS particles did not interact with MNPs *(Fig. 2B)*. If MNPs had interacted with the "broken" end of fragmented virions, TMVUSs, both ends of which are associated with MNPs, would statistically have occurred in the solution. However, this type of complexes was not detected.

Each TMV CP subunit is known to contain an RNA binding site that interacts with three nucleotides of the viral RNA. This CP–RNA interaction is observed when a guanine residue occurs in the third position of the binding site [15, 16]. Guanine residues are absent among the 69 first nucleotides of TMV RNA [17]. Therefore, the CP–RNA interaction is weak throughout the first 50–60 nucleotides, which may affect the surface charge distribution of the TMV virion.

**Fig. 3:**
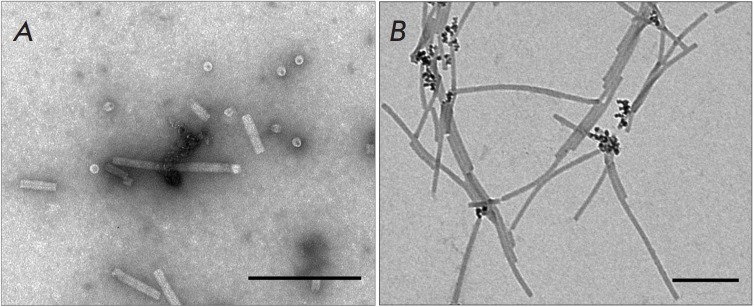
MNPs lack specific affinity to the ends of TMV CP repolymers (*A)*. Formation of native TMV–MNP complexes during preparation of TMV CP repolymers, control (*B)*. Scale bars are 200 nm

To elucidate the role of individual virion components in the formation of an increased negative charge area at one end of the TMV virus particle, we prepared TMV CP repolymers (virus-like particles, VLPs) with a helical structure similar to that of the virion, but lacking RNA [18]. An analysis of the complexes produced upon incubation of TMV repolymers with MNPs showed that MNPs either did not interact with TMV VLPs or bound to the entire surface of the repolymers (*Fig. 3A*). No complexes between MNPs and TMV VLP ends were observed. Because the binding of repolymers to MNPs occurred at pH 5.6 (the condition for TMV repolymer assembly), the TMV–MNP complexes obtained under the same conditions were used as controls. At pH 5.6, MNPs were found to interact also with only one end of the TMV virions *(Fig. 3B)*.

Therefore, the negative charge is uniformly distributed on the surface of VLPs obtained by polymerization of the TMV CP in the absence of RNA and is not localized at one of the ends, in contrast to the virion. RNA is likely to contribute significantly to the formation of an increased negative charge density area at one end of the native TMV.

To assess the contribution of viral RNA to the surface charge formation, TMV virions were treated with two nucleases: RNase A and micrococcal nuclease.

The ability of RNases to affect the TMV virion RNA was analyzed in preliminary experiments. Virions were treated with the nucleases. Then, RNA was isolated and analyzed by electrophoresis in 1% agarose gel. RNase A hydrolyzed the nucleic acid to fragments with an electrophoretic mobility comparable to that of the tRNA used as a co-precipitator or less (data not shown).

Micrococcal nuclease, as shown previously [19], does not hydrolyze RNA in the virion. However, it cannot be ruled out that at one of the virion ends, where the increased negative charge density area is located, the CP packaging is less dense, or the interaction between CPs and RNA is weaker throughout the first 50–60 nucleotides [17, 20], and small TMV RNA fragments within this area may be cleaved out. The analysis method used did not allow us to detect these changes.

**Fig. 4:**
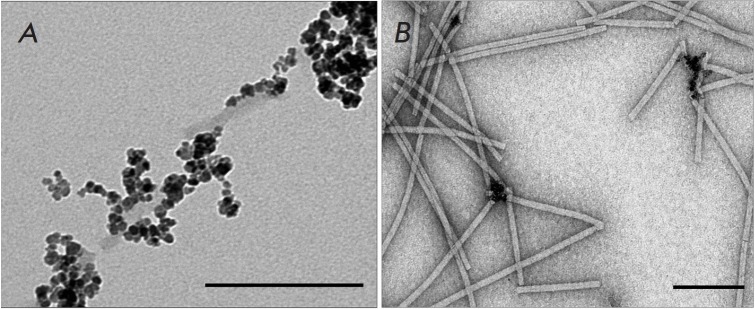
Analysis of the interaction between MNPs and TMV virions treated with RNase A (*A*) and micrococcal nuclease (*B*). Scale bars are 200 nm

There was no predominant affinity of magnetic nanoparticles to the ends of the virions treated with RNase A; the interaction occurred over the entire surface of the viral particles *(Fig. 4A)*. The surface charge of TMV virions containing degraded RNA was evenly distributed, as in TMV VLPs (*Fig. 3A*). However, an unusual pattern was observed upon the incubation of MNPs with MN-treated virions. Magnetic nanoparticles were not only located at the virion ends but also interacted with the entire surface of virions *(Fig. 4B)*. One may assume MN to be able to partially hydrolyze an RNA fragment that forms an increased negative charge density area. This is consistent with a model of the cotranslational TMV disassembly mechanism [21]. Most likely, the TMV virion end interacting with the MNP contains the 5’-end of TMV RNA.

Further investigation of the virion surface charge distribution was carried out on viruses with a flexible filamentous virion, which belong to the *Potexvirus *genus: potato virus X (PVX) and alternanthera mosaic virus (AltMV). We did not find any published data on the charge distribution on the AltMV or PVX surface; only the PVX isoelectric point is known (pI of 4.4).

Like the TMV CP, the AltMV CP is able (in the absence of RNA) to form *in vitro *stable extended particles similar in length and morphology to AltMV virions – AltMV virus-like particles (AltMV VLPs) [12].

**Fig. 5:**
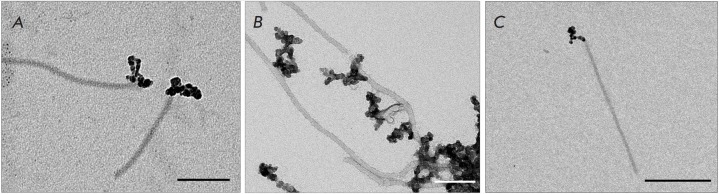
Complexes of MNPs with AltMV virions (*A*), AltMV VLPs (*B*), and PVX virions (*C*). Scale bars are 100 (*A, B*) and 200 (*C*) nm

As seen from *Figure 5*, MNPs, as in the case of TMV, form complexes with AltMV, binding to one end of the native virion (*Fig. 5A*).

An analysis of MNP–AltMV VLP complexes by electron microscopy showed that MNPs were distributed over the VLP surface. However, MNPs had no affinity to the VLP ends (*Fig. 5B*). Therefore, as in the case of TMV, the charge is unevenly distributed over the AltMV surface and the increased negative charge density area is also located at one end of the virion and is due to the presence of RNA. Similar results were obtained for PVX virions (*Fig. 5C*).

Earlier, we demonstrated that encapsulated PVX and AltMV RNAs, unlike TMV RNA, cannot be translated *in vitro* [22, 23].

Starting this study, we assumed that the previously discovered translational properties of RNA in TMV and some potexviruses [22, 24] would correlate with the charge density distribution on the surface of viral particles and that cotranslational TMV disassembly might be associated with an uneven distribution of the negative charge on the particle surface and with localization of the charge at the end comprising the 5’-end of the TMV RNA [20, 25]. At the same time, in potexviruses, whose viral RNA is not accessible to the ribosomes in the virion until specific translational activation, we thought we would observe a uniform charge distribution on the virion surface. Our data did not confirm this assumption.

**Fig. 6:**
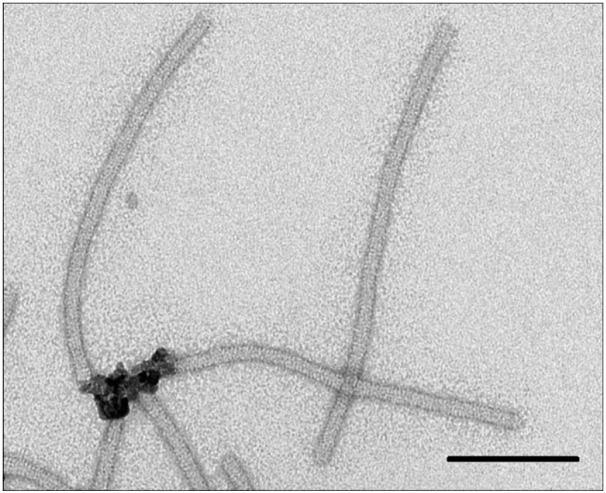
Complexes of MNPs with RNase A-treated PVX virions. The scale bar is 100 nm

The unexpected result was obtained for RNase A-treated PVX virions. Previously, treatment of PVX with RNases (A and T1) was shown to lead to RNA degradation into short segments (5 to 6 nucleotides) [26]. In this case, degraded RNA fragments remained in virions that were morphologically similar to filamentous PVX particles. In contrast to TMV, the interaction between MNPs and the ends of the PVX virions was detected (*Fig. 6*). Therefore, the surface charge of the PVX virions containing degraded RNA is unevenly distributed, like in native virions (*Fig. 5C*). It may be assumed that an RNA fragment located at the viral particle end remains bound to the CP and forms an increased negative charge density area. This result is consistent with the differences in the translational properties of encapsulated RNA in PVX and TMV virions.

Unlike TMV, the PVX CP is incapable of polymerization in the absence of RNA [27]. However, upon incubation with RNA *in vitro*, the PVX CP is able to form RNA-containing vRNPs and PVX CPs that have a helical "head," whose structure is identical to that of the protein helix of PVX virions, and a "tail"–CP-free RNA (one-tailed particles) [28].

**Fig. 7:**
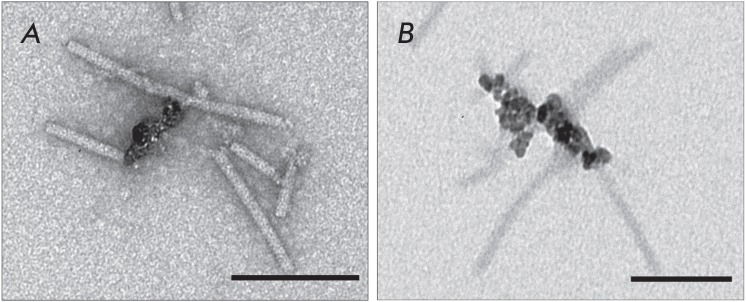
Complexes of MNPs with PVX vRNPs (*A*) and MN-treated vRNPs (*B*). Scale bars are 100 nm

Investigation of the interaction between magnetic nanoparticles and PVX vRNPs revealed binding of MNPs to CP-free RNA (vRNP tails) (*Fig. 7A*). The lack of interaction between MNPs and the vRNP head surface may be explained by the competitive binding of all available MNPs to free RNA. To prevent this, vRNPs were treated with MN. The PVX CP is known to cap the 5’-end of RNA upon vRNP treatment, and the 5’-terminal CP-encapsulated RNA segments in vRNPs retain their integrity and translational properties during treatment with MN [13]. An analysis of these complexes revealed that most MNPs, upon removal of free RNA, interact with the ends of MN-treated vRNPs (*Fig. 7B*), as in the case of native PVX (*Fig. 5C*).

Most likely, the end of viral particles, which interacts with MNPs, contains the 5´-end of RNA.

The experimental results are summarized in the *Table*.

**Table:** Mapping of the surface negative charge of helical plant viruses

Object/Treatment	TMV	AltMV	PVX
Virions	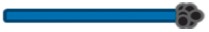	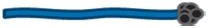	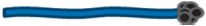
Ultrasound treatment	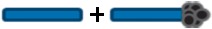		
VLP	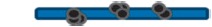	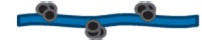	
RNase A	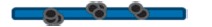		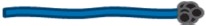
MN	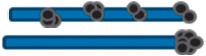		
vRNP			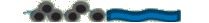
vRNP + MN			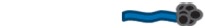

## CONCLUSIONS

In this study, the surface charge of TMV (Tobamovirus genus) and PVX and AltMV (Potexvirus genus) was mapped.

During the TMV assembly, the CP in the form of 20S discs is known to interact with an RNA site located at a distance of about 1,000 nucleotides from the 3’-end of the molecule (assembly origin) [18]. At the same time, the assembly of PVX virions begins directly at the 5’- end of the molecule; RNA interacts with CP monomers and dimers [27]. There is little data on the assembly of AltMV, but the assembly apparently occurs in the same way as in PVX [29]. It should be noted that in TMV, PVX, and AltMV, despite the fact that the assembly of virions occurs in different scenarios, the surface charge is distributed unevenly and an increased negative charge density area is located at one end of the virion. The key role in the formation of this area is apparently played by the 5’-end of viral RNA. Most likely, this may be explained by a less dense packing of the 5’-end of RNA in CP, which is required to initiate translation of RNA-dependent RNA polymerase at the early stages of infection of RNA-containing viruses with a positive genome.

## Abbreviations

TMV: tobacco mosaic virus
PVX: potato virus X
AltMV: alternanthera mosaic virus
CP: coat protein
VLP: virus-like particle
vRNP: viral ribonucleoprotein
MN: micrococcal nuclease
MNP: magnetic nanoparticle
TEM: transmission electron microscopy
